# A 3D‐Printed Self‐Adhesive Bandage with Drug Release for Peripheral Nerve Repair

**DOI:** 10.1002/advs.202002601

**Published:** 2020-10-19

**Authors:** Jiumeng Zhang, Yuwen Chen, Yulan Huang, Wenbi Wu, Xianming Deng, Haofan Liu, Rong Li, Jie Tao, Xiang Li, Xuesong Liu, Maling Gou

**Affiliations:** ^1^ State Key Laboratory of Biotherapy and Cancer Center West China Hospital Sichuan University Chengdu Sichuan 610041 P. R. China; ^2^ State Key Laboratory of Cellular Stress Biology Innovation Center for Cell Signaling Network School of Life Sciences Xiamen University Xiamen Fujian 361102 P. R. China; ^3^ Department of Urology Institute of Urology West China Hospital Sichuan University Chengdu Sichuan 610041 P. R. China; ^4^ Department of Neurosurgery West China Hospital Sichuan University Chengdu Sichuan 610041 P. R. China

**Keywords:** 3D printing, biomaterials, drug delivery, peripheral nerve repair, self‐adhesive bandages

## Abstract

Peripheral nerve injury is a common disease that often causes disability and challenges surgeons. Drug‐releasable biomaterials provide a reliable tool to regulate the nerve healing‐associated microenvironment for nerve repair. Here, a self‐adhesive bandage is designed that can form a wrap surrounding the injured nerve to promote nerve regeneration and recovery. Via a 3D printing technique, the bandage is prepared with a special structure and made up of two different hydrogel layers that can adhere to each other by a click reaction. The nanodrug is encapsulated in one layer with a grating structure. Wrapping the injured nerve, the grating layer of the bandage is closed to the injured site. The drug can be mainly released to the inner area of the wrap to promote the nerve repair by improving the proliferation and migration of Schwann cells. In this study, the bandage is used to assist the neurorrhaphy for the treatment of complete sciatic nerve transection without obvious defect in rats. Results indicate that the self‐adhesive capacity can simplify the installation process and the drug‐loaded bandage can promote the repairing of injured nerves. The demonstrated 3D‐printed self‐adhesive bandage has potential application in assisting the neurorrhaphy for nerve repair.

## Graphical abstract

A self‐adhesive bandage consists of two layers made of two clickable materials. This bandage can adhere itself via a click reaction. Due to big specific surface area of the grating layer and blocking by the outer layer, drug is mainly distributed at the injured site to promote nerve repair and the side effects would be reduced.

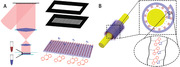

## 1 Introduction

Peripheral nerve injury (PNI) is one of common diseases resulting from trauma, tumors and other illnesses, which always leads to longstanding disability with loss of motor and/or sensory function.^[^ ^1^ ^]^ Unlike the central nervous system, the peripheral nervous system has pro‐regenerative factors, which enable it to regenerate itself after being injured.^[^ ^2^, ^3^ ^]^ However, the proper regeneration is limited and highly dependent on the type of injury, the size of the gap, the successful infiltration of cells, and the regeneration promoting factors at the site of injury. Only 50% patients could regain useful function by single neurorrhaphy treatment.^[^ ^4^ ^]^ With further research, it has been found that a favorable microenvironment could promote active pathophysiological response, such as developing regenerative phenotypes of neurons and promoting Schwann cells forming myelin sheath.^[^ ^2^ ^]^ Therefore, new treatment strategies should be developed to build a supportive environment for promoting the regenerative ability of injured peripheral nerve.

Pharmacological and molecular therapies are always combined with novel surgical intervention to regulate the nerve healing‐associated microenvironment.^[^ ^5^ ^]^ Some pharmaceuticals, including small molecules, peptides, hormones and growth factors, have been suggested as potential candidates to improve nerve regeneration.^[^ ^2^, ^6^, ^7^ ^]^ Melatonin has been demonstrated to accelerate the recovery of peripheral nerves by inhibiting oxidative stress and inflammatory response after trauma. It also triggers autophagy, accelerates the proliferation of regenerated nerve and reduces apoptosis.^[^ ^8^ ^]^ The XMU‐MP‐1 (4‐((5,10‐dimethyl‐6‐oxo‐6,10‐dihydro‐5H‐pyrimido[5,4‐b]thieno[3,2‐e][1,4]diazepin‐2‐yl)amino) benzenesulfonamide) is a reversible and selective Kinases MST1 and MST2 inhibitor that can block MST1/2 kinase activities to activate the downstream effector Yes‐associated protein (YAP) and promote cell growth. It has been used to promote the proliferation and migration of Schwann cells, thereby accelerating remyelination.^[^ ^9^ ^]^ However, the pharmaceutical treatment is facing the challenge how to precisely deliver drugs to the target site. Recently, pharmaceuticals were incorporated into nerve guidance scaffolds (NGSs) for local release to accelerate peripheral nerve repair.^[^ ^10^ ^]^ But parts of the drugs are usually released to the surrounding tissues, which would reduce drug concentrations at target sites and take the risk of side effects.

NGSs have been proved as an effective treatment in peripheral nerve repair.^[^ ^11^ ^]^ Enhanced by pharmaceuticals, the functionalized NGSs could regulate the biochemical environment to accelerate the reconnection of two stumps. But, the convenient NGSs face the problem of cumbersome installation processes: nerve conduits need to let the frail injured nerve pass through, and nerve wraps need to be sutured for fixation.^[^ ^12^ ^]^ To be implanted at delicate nerves, NGSs should be easily installable to avoid nerve damage during the surgery. Tao and his workmates designed a shape memory nerve conduit that can be compressed and recover into a long tube after surgery. This nerve conduit showed great effect in simplifying the installing process and promote peripheral nerve repair.^[^ ^13^ ^]^ Nerve wraps can be easily rolled around the injured nerves to form a size‐matched cavity. But it also needs suture or glue to fix the wraps.

In recent years, 3D printing technology, an emerging manufacturing method, has been widely applied in fabricating NGSs.^[^ ^14^ ^]^ This manufacturing method has a great advantage in programming materials and structures.^[^ ^15^ ^]^ A variety of well‐designed structures were fabricated in NGSs to accelerate nerve regeneration. The high precision 3D printing technology could incorporate structures within the NGSs lumen for potentially improving microstructural guidance.^[^ ^16^ ^]^ The customized nerve conduits based on medical image data could be installed at injured nerves perfectly.^[^ ^17^ ^]^ Nerve conduits could be lend branch structure to match the facial nerves.^[^ ^14^ ^]^ What's more, pharmaceuticals can be combined in NGSs by 3D printing technology and released in situ.^[^ ^18^ ^]^ By adjusting materials, structures and drug concentrations, researchers could control the release process of pharmaceuticals in the 3D printed products.^[^ ^19^ ^]^

**Figure 1:**
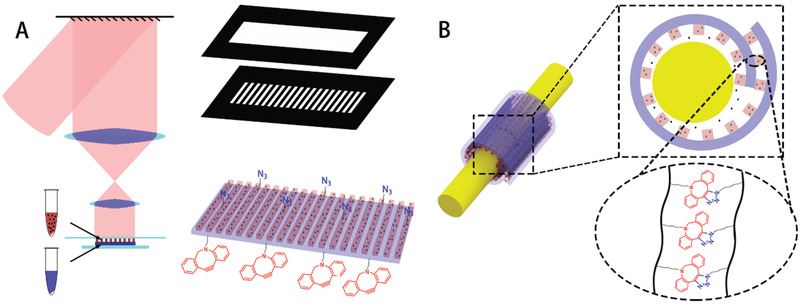
The 3D printed self‐adhesive drug‐loaded bandage surrounding a nerve and releasing drugs. A) Scheme for fabricating the self‐adhesive drug‐loaded bandage, a rectangle layer is printed by DBCO‐GelMA, then a grating layer is printed by N_3_‐GelMA combined with XMU‐MP‐1 nanoparticles. B) Scheme of the microenvironment that injured nerves are in, the bandage adhere itself by the click reaction of −N_3_ and –DBCO, the drugs loaded in the grating layer are directionally released to inner side of the wrap.

In this work, we 3D‐printed a biodegradable self‐adhesive bandage (SAB) made of a set of clickable functionalized monomers (azide modified gelatin methacrylate (N_3_‐GelMA) and dibenzyl cyclooctyne modified gelatin methacrylate (DBCO‐GelMA)), which can wrap injured nerves and release drugs directionally for nerves repair (**Figure** **1**A). Taking advantages of the flexibility of 3D printing technology, the set of monomers and nanodrug were programmed on the specific location to form the bandage that consisted of two layers with rectangle and grating structures respectively. Then, the biodegradable bandage can adhere itself to form a side‐close wrap and provide a protection for the injured nerve. Meanwhile, XMU‐MP‐1 nanoparticles were loaded in the grating layer which finally became the inner wall of the wrap attached to the injured nerve. XMU‐MP‐1 could be directionally released to the injured site continuously. Through electrophysiological assessment and histological examinations of the rat sciatic nerve transected models, it was verified that the self‐adhesive drug‐loaded bandages (SADB) can significantly benefit peripheral nerve regeneration and recovery.

## 2 Results

### 2.1 Synthesis of the Clickable Photopolymerizable Monomers

The biorthogonal reaction between azido groups (–N_3_) and cyclooctyne groups (–DBCO), i.e., strain‐promoted alkyne‐azide cycloaddition (SPAAC) without copper catalysis, would lend the polymers the capacity of self‐adhesion.^[^ ^20^ ^]^ The reaction is highly specific and the materials would not react with other materials or human tissues.^[^ ^21^ ^]^ Benefited by the properties of efficiency and catalyst‐free, this set of monomers is well suited for in vivo application. GelMA is a photopolymerizable material widely applied in light‐assistant 3D printing and tissue engineering.^[^ ^22^ ^]^ To fabricate the self‐adhesive bandages, a set of 3D printable self‐healing monomers (DBCO‐GelMA and N_3_‐GelMA, Figure S1A, Supporting Information) were synthesized by modifying gelatin. The synthetic process is divided into two steps. Firstly, NH_2_‐GelMA was synthesized by referring to the method of synthesizing GelMA.^[^ ^17^ ^]^ To optimize the ratio of MA to N_3_ or MA to DBCO, the methylacrylic acid anhydride (MAA) should be reduced to 0.06 mL g^−1^ gelatin for leaving part amino groups for the modification of N_3_ and DBCO. Then, N‐hydroxysuccinimide functionalized N_3_ (N_3_‐NHS) or DBCO‐NHS and trimethylamine were added to NH_2_‐GelMA solution to obtain the set of clickable functionalized monomers. The nuclear magnetic resonance (NMR) and Fourier transform infrared spectrometer (FTIR) analyses verified that the functional groups were introduced into GelMA successfully (Figure S1B,C, Supporting Information).

To ensure the ratio of MA to N_3_ or MA to DBCO, the substitution degrees were tested. The substitution degree of MA of NH_2_‐GelMA was about 71.9% ± 3.0%. The substitution degrees of N_3_ and DBCO in monomers were 23.8% ± 2.5% and 27.2% ± 2.6%, respectively (Figure S1D, Supporting Information). The abilities of photopolymerization and self‐adhesion were verified by testing the gel‐ability of the monomer solutions (Figure S1E, Supporting Information). After exposing for 10 s by 405‐nm ultraviolet (UV) light, DBCO‐GelMA and N_3_‐GelMA solutions (5% w/v) containing photoinitiator lithium phenyl‐2,4,6‐trimethyl‐benzoylphosphinate (LAP) (1% w/v) polymerized into hydrogels respectively (Figure S1Ea,b, Supporting Information). The hydrogel was formed after the solutions of DBCO‐GelMA and N_3_‐GelMA were mixed together (Figure S1Ec, Supporting Information), indicating that DBCO and N_3_ in the monomers could reacted via SPAAC. Taking together, the synthesized DBCO‐GelMA and N_3_‐GelMA can work as a 3D printable self‐adhesive ink.

### 2.2 Self‐Adhesive Capacity of the Hydrogels

The self‐adhesive capacity is the foundation that nerve bandages can be installed without additional suturing during surgeries. The adhesive capacity between N_3_‐GelMA and DBCO‐GelMA hydrogels was tested as follows. Firstly, N_3_‐GelMA hydrogel block (N_3_‐block) and DBCO‐GelMA hydrogel block (DBCO‐block) could adhere together well. They did not separate even though they were deposited obliquely at the angel of 90° (Figure S2A, Supporting Information). Next, a red dye (Vb12) did not leak out of the container assembled by adhering DBCO‐block wall and N_3_‐block bottom (Figure S2B, Supporting Information), indicating that the blocks could stably adhere to each other. Finally, referring to the method for measuring the shear viscosity of liquid surface, the adhesive intensity between DBCO‐block and N_3_‐block was test.^[^ ^23^ ^]^ The blocks were overlapped to obtain three groups, i.e., DBCO‐block to N_3_‐block (C–N) group, DBCO‐block to DBCO‐block (C–C) group, and N_3_‐block to N_3_‐block (N–N) group. The loss moduli of N–N, C–C, and N–C groups were 15.54, 3.20, and 19.97 Pa, respectively (Figure S2C, Supporting Information). The loss modulus of C–N group is much higher than that of N–N and C–C groups, indirectly indicating that the adhesion between the two blocks was enhanced by the crosslinking between –N_3_ and –DBCO.

### 2.3 Preparation of the Self‐Adhesive Bandage

**Figure 2:**
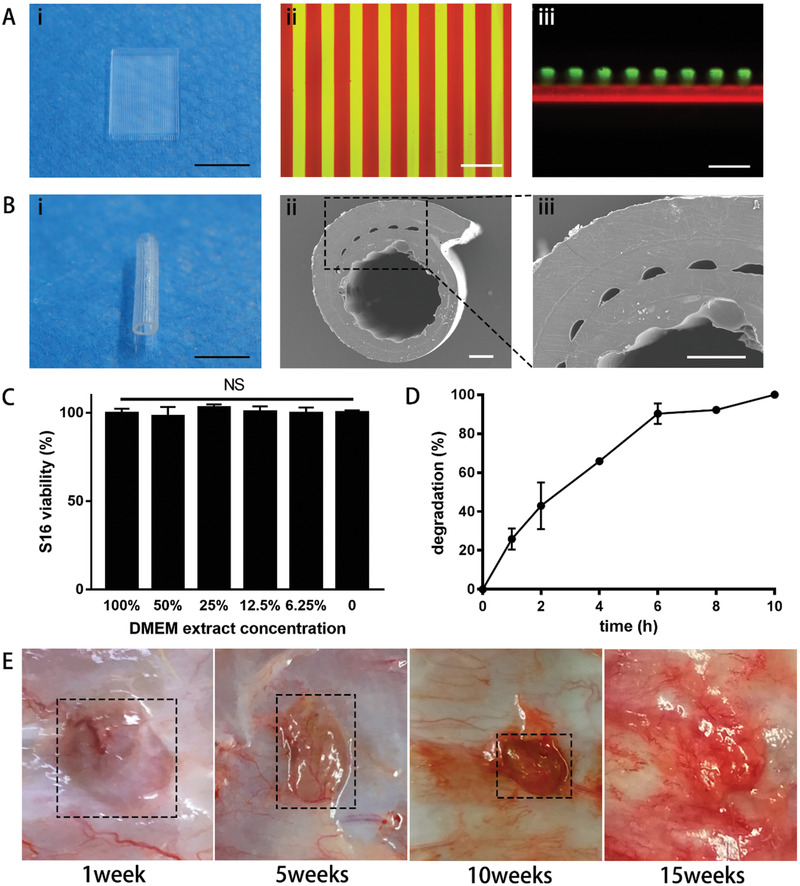
Structure and biocompatibility characterization of the self‐adhesive bandages. A) 3D printed self‐adhesive bandage before rolling, camera photo (bar = 5 mm); fluorescence images of the top view and side view of the bandage, red (rhodamine) and green (FITC) showed the rectangle layer and the grating layer in turn (bar = 500 µm). B) The bandage after rolling and self‐adhering, camera photo (bar = 5 mm); SEM images showed the self‐adhered part (bar = 200 µm). C) S16 viabilities in DMEM extract of the bandage (100%, 50%, 25%, 12.5%, 6.25%, 0). These data are presented as mean ± standard deviation, *n* = 3, one‐way ANOVA, NS. D) Degradation rate of self‐adhesive bandage in collagenase I (0.5 mg mL^−1^). These data are presented as mean ± standard deviation, *n* = 3. E) Degradation degree of self‐adhesive bandage in the back of the SD rats, hydrogels almost disappeared after 15 weeks.

Using the photopolymerizable clickable monomers, we fabricated the self‐adhesive bandage as it shown in Figure 1. In this work, a DLP‐based 3D printing technology was employed to fabricate the bandages with the double‐layer structure. Via this technique, the first layer made of DBCO‐GelMA was printed by projecting a rectangle pattern. Then another ink containing N_3_‐GelMA and drug‐loaded nanoparticles was added to replace the previous ink. By projecting a grating pattern, corresponding structure was formed upon the first layer. It could be seen clearly that there were some streaks on the layer of the hydrogel (**Figure** **2**Ai). Some researchers have found that the grating structure can increase specific surface area and accelerate the drug release.^[^^]^ The microstructures of each layer were observed by fluorescence microscope. Two fluorochrome, rhodamine (red) and fluorescein isothiocyanate isomer (FITC) (green), were used to label two blocks respectively. In the top view, the grating thickness and interval were about 130 and 180 µm (Figure 2Aii). In the cross view, the thicknesses of the two layers were about 450 and 320 µm (Figure 2Aiii). The 3D‐printed thickness of each layer was 250 µm. The two layers made from DBCO‐GelMA and N_3_‐GelMA had different swelling ratio, which leaded to different increase in thicknesses.

After rolling and self‐adhering, the bandage became a wrap structure (Figure 2Bi). Then, the wrap was observed by scanning electron microscope (SEM) (Figure 2Bii,iii). The thickness and interval of the streaks were 92 and 82 µm. The thicknesses of the two layers were 139 and 110 µm respectively. Due to the shrinking during gradual dehydration and drying, the objects seen in SEM were smaller than in fluorescence microscope. Compared with the first layer, the second grating layer shrunk more seriously and its thickness was smaller. More importantly, as the arrows showed, the junctions were adhered obviously. After the bandage was rolled, the drug‐loaded layer was placed to the inner side. Thus, the bandages could be fixed at injury sites and release drugs to the injured nerve intensively.

To verify its potential of in vivo application, we evaluated the biocompatibility and biodegradability of the bandage. N_3_‐GelMA and DBCO‐GelMA were verified to be harmless materials via a series of tests in vitro and in vivo. Firstly, the self‐adhesive bandage was immersed in Dulbecco's modified eagle medium (DMEM) for 72 h at 37 °C to obtain its extract. The methyl thiazolyl tetrazolium (MTT) assay was used to investigate the cytotoxicity of the extract to Schwann cells in vitro. The viabilities of S16 (Schwann cells) in extract solutions at different concentrations (100%, 50%, 25%, 12.5%, 6.25%, 0) were 99.72% ± 2.45%, 97.72% ± 5.43%, 102.7% ± 1.99%, 100.3% ± 3.24%, 99.57% ± 3.28%, 100% ± 1.224% (Figure 2C). There was no significance between the cell viabilities of each group. The biodegradability of the self‐adhesive bandages was studied in vitro and in vivo respectively. The collagenase I solution (0.5 mg mL^−1^) was used for degradation test of the self‐adhesive bandage. About 90% weight of the bandage hydrogel was lost after 6 h. Until 10 h, the bandage hydrogels were destroyed completely (Figure 2D). Finally, the bandages were implanted subcutaneously on the back of Sprague Dawley (SD) rats for in vivo degradation test. The states of the bandages were observed at 1 week, 5 weeks, 10 weeks, and 15 weeks (Figure 2E). At the first week, there was a lump hydrogel on the back of the rats. This was because the hydrogel was too soft to crumple into a lump. The lump of hydrogel became smaller and smaller as time goes on. After 15 weeks, it disappeared completely. Meanwhile, by common observation, there was no visual inflammation around the implants all the time. The self‐adhesive bandages exhibited good biocompatibility and biodegradability and can be implanted in vivo.

### 2.4 Gradient Release of Nanoparticle Drugs

**Figure 3:**
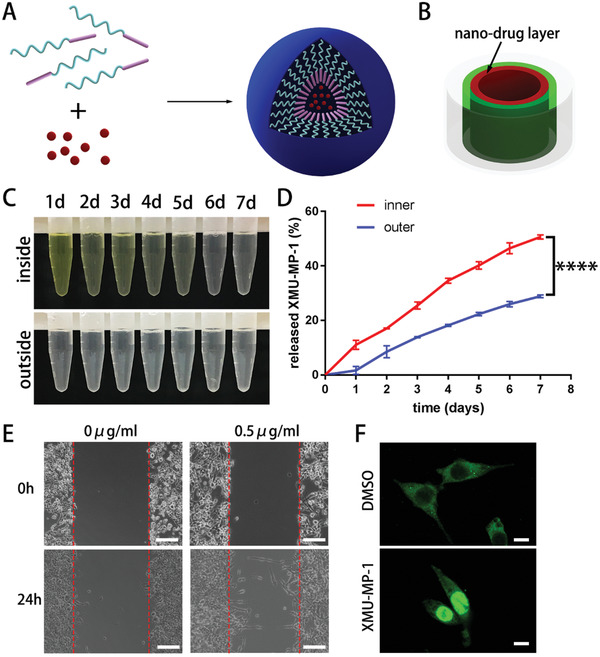
Preparation of XMU‐MP‐1 loaded nanoparticles and in vitro demonstration of drug release. A) MPEG‐PCL formed a core–shell structure: a hydrophobic PCL core and a hydrophilic PEG shell, the nanoparticles encapsulated drugs in the core by self‐assembling. B) Scheme of drug release model simulated the release in vivo, green is the outside hydrogel and red is the inside hydrogel containing nanoparticles, both of them are in a container (gray). C) The collected curcumin solution in the inside and outside of the model respectively from the first day to the seventh day. D) The accumulated quantities of XMU‐MP‐1 released to inside and outside from the first day to the seventh day, the quantity of the inner XMU‐MP‐1 was significantly higher than that of the outer XMU‐MP‐1. These data are presented as mean ± standard deviation, *n* = 3, Student's *t*‐test, ****p < 0.0001. E) The wound‐healing assay of S16 treated by 0.5 µg mL^−1^ XMU‐MP‐1 (bar = 200 µm). F) Representative immunofluorescence images showed the distributions of YAP in S16 after treated by 0.5 µg mL^−1^ XMU‐MP‐1 (bar = 200 µm).

Besides neurorrhaphy treatment and simple NGSs, drug‐assisted treatment has been widely used in repairing PNI. XMU‐MP‐1 can regulate YAP that was selectively expressed in neural stem cells and Schwann cells and has been used to treat PNI. To improve its water solubility and increase release time, XMU‐MP‐1 was encapsulated into monomethoxy poly(ethylene glycol)‐poly(*ε*‐caprolactone) (MPEG‐PCL) nanoparticles to enhance the water solubility and extend the releasing time. Through a self‐assembled process, MPEG‐PCL formed a core–shell structure and encapsulated drugs in the core (**Figure** **3**A). XMU‐MP‐1 nanoparticles dispersed uniformly in water and caused Tyndall Effect (Figure S3B, Supporting Information). The mean zeta potential and size of the XMU‐MP‐1 nanoparticles were −3.3 mV and 74 nm (Figure S3C, Supporting Information), respectively. Moreover, the transmission electron microscopy (TEM) image showed that these XMU‐MP‐1 nanoparticles were monodisperse and had the mean particle size of about 50 nm (Figure S3A, Supporting Information). TEM presented the dry condition of the nanoparticles and the dynamic light scattering showed the hydrodynamic diameter of nanoparticles which dispersed in water, so the mean particle sizes presented difference.

The specific structure of the bandage is designed to control drug centrally release to the wrapped nerve. To measure the difference of inner and outer drug concentrations of the wrapped bandage ex vivo, a drug release model was designed and fabricated to simulate the release of XMU‐MP‐1 loaded in the bandage (Figure 3B). The double‐layer consisted of inside nanodrug layer and outside blocked layer. In this release model, the drugs released to inside and outside could be separated and collected. Firstly, to increase the visibility, a water‐soluble dye (curcumin) was used as a model drug to stimulate XMU‐MP‐1 release (Figure 3C). By common observation, the yellow of the solutions from the inner side was much darker than that from outer side. It meant that inner curcumin concentrations were higher than outer concentrations. Then, XMU‐MP‐1 was also loaded, released, and collected via this model. Based on the ultraviolet absorption, the quantities of the XMU‐MP‐1 (an absorption peak at 303 nm) were calculated and compared (Figure 3D). The inside XMU‐MP‐1 was obviously higher than that in the outside area. Due to the rational structure design, the diffuse process of the released drug was prevented by the outer hydrogel layer. In addition, the release process was sustained and drugs could work continuously. The release result indicated that the bandages could control the direction of drug release and reduce drug concentrations at surrounding tissues.

In previous study, XMU‐MP‐1 has been used for treating PNI.^[^ ^9^ ^]^ Herein, we explored how XMU‐MP‐1 could promote the proliferation and migration of Schwann cells to accelerate peripheral nerve repair. By the analysis of MTT, XMU‐MP‐1 was showed to promote S16 cells proliferation in a certain range of concentration. The cell proliferation rates at the concentration of 0.03125, 0.0625, 0.125, 0.25, and 0.5 µg mL^−1^ were increased by 1.30% ± 1.30%, 1.30% ± 1.00%, 2.42% ± 0.40%, 3.42% ± 0.60% and 4.10% ± 0.77%, respectively (Figure S3D, Supporting Information). Wound healing experiment revealed that XMU‐MP‐1 could also promote S16 cells migration at the concentration of 0.5 µg mL^−1^ (Figure 3E). Some studies have showed that XMU‐MP‐1 treatment could induce predominant nuclear localization of YAP in cells.^[^ ^25^ ^]^ Furthermore, we verified how XMU‐MP‐1 influenced Schwann cells. Via immunofluorescence, the distributions of YAP in S16 cells were shown in Figure 3F. In the immunofluorescence images, YAP was mainly located in cell nucleus when S16 were treated by XMP‐MP‐1 (0.5 µg mL^−1^). But little YAP was observed in cell nucleus of the control group. The previous study has shown that the increasing of YAP nuclear translocation could leading to the upregulation of YAP target genes: cysteine‐rich angiogenic inducer 61 (CYR61) and connective tissue growth factor (CTGF),^[^ ^9^ ^]^ which explained the potential mechanism of how XMU‐MP‐1 promoted the proliferation and migration of Schwann cells. Therefore, the XMP‐MP‐1 nanoparticles have the potential for nerve repair in vivo.

### 2.5 Surgery and Functional Evaluation In Vivo

**Figure 4:**
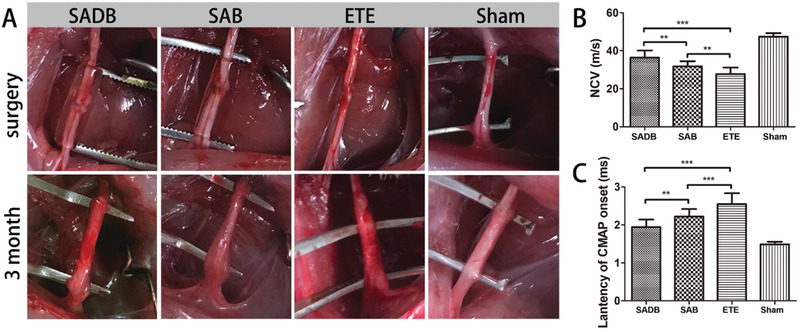
Functional evaluation of the biodegradable self‐adhesive bandages in vivo. A) Conventional observation of the operated nerves and regenerated nerves. B) The NCV value of the regenerated nerves. C) The latency of CMAP onset of the regenerated nerves. These data are presented as mean ± standard deviation, *n* = 6, one‐way ANOVA, **p < 0.01, ***p < 0.001.

To evaluate the function of the self‐adhesive drug‐loaded bandages for peripheral nerve repair, we used the sciatic nerve transection models in SD rats (**Figure** **4**A). The rats were divided into four groups according to different treatment methods, including SADB group, SAB group, only end‐to‐end (ETE) neurorrhaphy group, and sham‐operated (Sham) group. When the nerves were sutured by neurorrhaphy, the bandages were taken to wrap the nerves with the grating layer inside. Because of the surface tension and click reaction, the 3D‐printed hydrogel bandage could roll around the nerve and adhere itself, which could save much time during the surgery. Three months post‐surgery, the regenerated sciatic nerve was exposed and evaluated. Here, several evaluation strategies, including electrophysiological analysis, nerve morphological analysis and amyotrophy analysis, were applied to evaluate the regeneration and recovery of the nerves. It showed that the distal and proximal sites were reconnected, and no neuroma was formed at the sutured sites in all groups (Figure 4A). The electrophysiology was used to test the functional recovery of the injured peripheral nerve. The mean nerve conducting velocities (NCV) of SADB, SAB, ETE and Sham group were 36.7 ± 3.8, 31.5 ± 3.0, 27.9 ± 3.4 and 47.5 ± 1.9 m s^−1^, respectively (Figure 4B). The mean latencies of the compound motor action potential (CMAP) onset of SADB, SAB, ETE and sham group were 1.93 ± 0.20, 2.23 ± 0.22, 2.55 ± 0.29, and 1.49 ± 0.07 ms, respectively (Figure 4C). The faster NCV and shorter latency indicated a better nerve regeneration and functional recovery in SADB and SAB groups.

**Figure 5:**
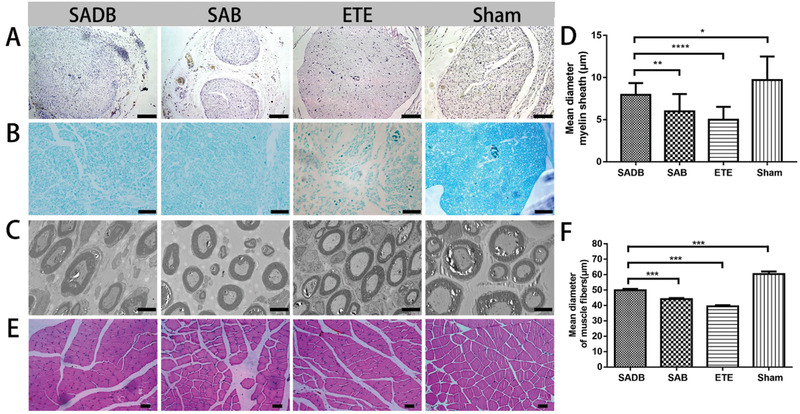
Histological analysis of repaired nerves and surrounding muscles. A) H&E of repaired nerves (bar = 100 µm). B,C) Axon and myelin sheath in light microscope (bar = 50 µm) and TEM (bar = 50 µm). D) Diameter of myelin sheath. These data are presented as mean ± SEM, *n* = 16, one‐way ANOVA, *p < 0.05, **p < 0.01, ****p < 0.0001. E) H&E of muscles (bar = 50 µm). F) Diameter of muscle fibers. These data are presented as mean ± SEM, *n* = 90, one‐way ANOVA, ***p < 0.001.

After electrophysiological analysis, the regenerated nerves were immediately dissected for morphological analysis. Samples were processed by hematoxylin & eosin (H&E) staining and Luxol fast blue (LFB) staining, and observed in optical microscopy and TEM. Representative images of all groups are shown in **Figure** **5**A. The H&E images in all groups showed that regenerated nerves were organized and lacked scar tissues. Then, we analyzed the status of the myelin sheath (Figure 5B,C). The LFB staining showed that the myelin sheath in SADB group was bigger and more intensive than in SAB and ETE groups. In TEM images, the myelin sheath was observed clearly. The diameters of myelin sheath in SADB, SAB, ETE and sham groups were 7.95 ± 1.39, 5.99 ± 2.05, 5.00 ± 1.51, and 9.71 ± 2.79 µm, respectively (Figure 5E). The self‐adhesive bandages could accelerate the regeneration of myelin sheath to promote the nerve repairing. Meanwhile, adding XMU‐MP‐1 could further improve the therapeutic effect. It was consistent with the result of electrophysiological analysis. To assess the amyotrophy, the diameters of muscle fibers were compiled. The H&E images of the gastrocnemius showed that the muscles atrophied in different degrees (Figure 5E). Compared with sham group (60.45 ± 6.33 µm), the diameters of muscle fiber in SADB (49.91 ± 8.28 µm), SAB (44.16 ± 6.57 µm) and ETE (39.44 ± 14.84 µm) group decreased at different degrees (Figure 5F). It indicated that the SADB and SAB treatment could accelerate the reconnected nerves repairing and inhibit the muscular atrophy.

## 3 Discussion

The NGSs, which showed certain effects for nerve repairing, have been widely used in PNI.^[^ ^26^ ^]^ The NGSs were designed to entangle the injured nerves and provide a beneficial microenvironment for the neurons and Schwann cells. Incorporated with pharmaceuticals, the NGSs showed better treating efficiency, but how to precisely release drugs was a difficult problem. Meanwhile, NGSs faces a number of other challenges in its application. The size of nerve conduit usually mismatches with the injured nerve and the installation process of NGSs needs to be very careful to avoid injuring the nerve again. In this work, we firstly used the photopolymerizable clickable monomers to 3D‐print the self‐adhesive bandage for promoting peripheral nerve regeneration. This drug‐loaded bandage could be easily implanted to wrap an injured nerve for protecting it and directionally releasing drugs to regulate the nerve healing‐associated microenvironment. By a DLP‐based 3D printing technology, the set of clickable monomers were made into the upper and lower surfaces of the bandages respectively. Via the click chemistry, the biodegradable bandage could adhere itself without sutures or glue during the surgery. So, the self‐adhesive bandage could simplify the installment process of NGS and avoid other cumbersome operations. Most importantly, by constructing rational structures within the self‐adhesive bandage, the release of nanomedicine contained within the bandage could be controlled and the injured nerves could be effectively treated.

Despite the advantages of NGSs, they are facing the challenges, including size mismatching and difficult installation. The conventional nerve conduits had fixed sizes and could not change after being fabricated, which usually lead to the mismatch between conduits and nerves. Some studies have found that the matching degree of nerve conduits was closely associated with the treatment effect. Poorly fitted nerve conduit resulted in tube collapse, poor nerve regeneration, and decreased muscle re‐innervation.^[^ ^27^ ^]^ In our previous study, in order to obtain a conduit with similar shape and size to the injured nerve, Hu et al. fabricated the nerve conduit based on the data of nerve magnetic resonance imaging (MRI) by 3D print technology.^[^ ^17^ ^]^ Nerve wraps can wrap injured nerves and form suitable inner cavities. But they are required sutures or glue to secure them after rolled around the sutured nerves.^[^ ^28^ ^]^ The additional operation is laborious and may take the risk of secondary injury. Therefore, we improved conventional nerve wraps and fabricated a self‐adhesive bandage to solve these problems. The bandage could wrap injured nerves as band‐aid sticking skin and protecting wound by adhering itself. Self‐healing, which requires rebonding of a material to its original shape/condition, was considered to fabricate the self‐adhesive bandage.^[^ ^29^ ^]^ To wrap an injured nerve during the surgery, the bandage was required to adhere itself rapidly and specifically. The set of clickable monomers contain the functionalized groups of N_3_ and DBCO that can react efficiently and specifically without catalyzer via SPAAC.^[^ ^30^ ^]^ This reaction is biorthogonal and would not interfere with native biochemical processes in living systems.^[^ ^31^ ^]^ The in vitro and in vivo analysis verified the biocompatibility and biodegradability of the clickable monomers. These monomers could be applied to fabricate the safety and efficient self‐adhesive bandage for in vivo application.

Pharmacological enhancement of regeneration played an important role in increasing functional recovery of injured nerves. However, the drug concentrations must be appropriate for accelerating nerve regeneration and recovery effectively.^[^ ^7^, ^32^ ^]^ Suzuki et al. developed a novel electrospun nanofiber sheet that was incorporated with methylcobalamin (MeCbl) and could locally release the drug with a high concentration to the site of injured nerve.^[^ ^33^ ^]^ NGS is implanted right where the injured nerve is, and can delivers the drug locally. So, it can act as a proper drug delivery system for peripheral nerve repair. In our previous study, we have encapsulated adipose stem cells and RGFP966 in the nerve conduits.^[^ ^10^, ^17^ ^]^ In this work, an inhibitor, XMU‐MP‐1, which can block Mst1/2 activity was used to promote nerve regeneration and recovery. This drug has been proved to accelerate liver repair and regeneration.^[^ ^25^ ^]^ This work verified that XMU‐MP‐1 could induce predominant nuclear localization of YAP and promote proliferation and migration of Schwann cells. The Schwann cells can secrete nerve growth factor and form myelin sheath during the process of nerve repairing.^[^ ^2^ ^]^ To improve its water solubility and increase release time, XMU‐MP‐1 was encapsulated in MPEG‐PCL nanoparticles before mixed in 3D printed ink. Moreover, the XMU‐MP‐1 nanoparticles were loaded in the inside layer of the NGS. Blocking by the outside layer, only a few drugs would be released to the surrounding tissues. Thus, the directional releasing process could provide enough drug concentration for repairing injured nerve and reduce side effects to surrounding tissues.

3D printing technology provides a manufacturing method to fabricate NGSs that had complex structures and was incorporated with pharmaceuticals.^[^ ^11^, ^34^ ^]^ The DLP‐based 3D printing technique can print one layer with a high‐resolution pattern in several seconds.^[^ ^35^ ^]^ The self‐adhesive bandage is a two‐layer membrane composed of two different materials respectively. The DLP‐based 3D printing technology is very flexible and supports changing inks according to the materials requirements. So, it is an appropriate manufacturing method to fabricate this membranous bandage. Via this technology, we fabricated the rectangle layer and grating layer of the self‐adhesive bandage with DBCO‐GelMA and N_3_‐GelMA respectively. Then, the click reaction can only occur between upper and lower surfaces, which can avoid sticking between the same layers. When the bandage encased the injured nerve, the grating layer encapsulating XMU‐MP‐1 nanoparticles is abutting to the injured site. The 3D‐printed grating structure has a big specific surface area for drug release. The outer layer hindered the drug release at the same time. Thus, drugs could be released to the injured site intensively and continuously. The in vivo experiment results showed that the self‐adhesive bandage was benefit to promote nerve regeneration and functional recovery.

## 4 Conclusion

In this work, we demonstrated a 3D printed self‐adhesive drug‐loaded bandage for repairing peripheral nerve injures. This bandage could be readily installed by adhering itself via the click reaction. The spatial drug release of this bandage was benefit to improve the therapeutic efficiency and reduce the potential side effects. The in vivo experimental data showed that the bandages could effectively promote the regeneration and recovery of nerves. The described drug‐loaded bandage has potential application in peripheral nerve repair, which could lead to the development of future biomaterials for nerve repair.

## 5 Experimental Section

##### Synthesis and Characterization of Photopolymerizable Clickable Monomers

1 g type A gelatin (175 bloom) derived from porcine skin tissue was dissolved in 10 mL 0.25 m CB buffer (7.95 g sodium carbonate and 14.65 g sodium bicarbonate in 1 L distilled water). Subsequently, 60 µL methacrylic acid was added to the gelatin solution under magnetic stirring at 500 rpm. The reaction proceeded at 50 °C for 3 h, and then the pH was readjusted to 7.4 to stop the reaction. After being dialyzed, filtered, and lyophilized, the samples were stored at −20 °C for further use. NH_2_‐GelMA was dissolved in water, with stirring at 60 °C and then cooling down to 30 °C. Next, DBCO‐NHS or N_3_‐NHS and trimethylamine were added; the mixed solution was stirred for 24 h at 30 °C. The reaction mixture was then dialyzed against deionized water for 3 days at 30 °C. The solution was subsequently lyophilized to give the product as a white solid.

TNBS assay was performed to quantify the substitution degree. Briefly, gelatin, NH_2_‐GelMA, DBCO‐GelMA, and N_3_‐GelMA samples were separately dissolved at 1.6 mg mL^−1^ in 0.1 m sodium bicarbonate buffer. Then, each sample solution was mixed with 0.01% TNBS solution (in 0.1 m sodium bicarbonate buffer) both at 0.5 mL and then was incubated for 2 h. Next, 0.25 mL of 1 m hydrochloric acid and 0.5 mL of 10% w/v sodium dodecyl sulfate (SDS) were added to stop the reaction. The absorbance of each sample was measured at 335 nm. The glycine standard curve was plotted to determine the primary amino groups, with sample solutions prepared at 0, 8, 16, 32, 64 µg mL^−1^.

##### Self‐Adhesive Ability Test of the Clickable Hydrogel

Two hydrogel blocks (diameters are 5 and 10 mm) were printed with N_3_‐GelMA and DBCO‐GelMA respectively. The little one was stacked upon the other one for 5 min. Then, the blocks were deposited obliquely at the angel of 90° to verify if they were assembled together. To characterize the tightness of the self‐assembled part, an assembled container was fabricated. The wall and bottom of the container were made from N_3_‐GelMA and DBCO‐GelMA, respectively. After assembling, dye liquor (Vb12 aqueous solution, 0.5 wt%) was dumped into the container. The dye liquor was observed for whether it flowed out from the assembly joint.

The self‐adhesive strength of the clickable hydrogel was evaluated by measuring the loss modulus of overlapped blocks. The size of measured samples was required to be 8 mm diameter and 1 mm thin. The N_3_‐GelMA and DBCO‐GelMA blocks were fabricated as a half thickness (0.5 mm) of the samples. Then, the blocks were overlapped and put into a rheometer (Rheostress 6000) to measure their loss moduli at 0.1 Hz frequency and 1% strain limit.

##### Preparation of Self‐Adhesive Bandages

The preparation of XMU‐MP‐1 nanoparticles is described as follows: First, 1 mg of XMU‐MP‐1 and 99 mg MPEG‐PCL copolymer were co‐dissolved in 200 µL of DMSO. Then the solution was dropped into the 9.8 mL of distilled water with gentle stirring. During the stirring process, MPEG‐PCL copolymer could self‐assemble into a core–shell structure. A DLP‐based 3D printer was used for fabricating the bandages. Before 3D printing, the 3D digital model of the bandage was designed and loaded into the DLP‐based 3D printer. The first layer was printed with a rectangle pattern by the ink (10% DBCO‐GelMA). The second layer was printed with a grating pattern by the ink was (10% N_3_‐GelMA with or without 1% XUM‐MP‐1 nanoparticles). Each layer was 250 µm. Each ink was mixed with 1% photoinitiator (LAP).

To observe the bandages clear, FITC (0.5 mg mL^−1^) and Rhodamine (0.5 mg mL^−1^) were added into the printed inks severally. After printing, the top and side views of the bandage were observed by a fluorescence microscope. The bandage was rolled around a 1.5 mm diameter pillar to simulate its state after being implanted. After gradual dehydration (in 15%, 30%, 45%, 60%, 75%, 90%, 100% alcoholic solution), critical point drying, and Pt/C‐shadowing, the bandage was visualized by SEM.

##### Release Experiment

The release model could separate and collect the released drug of the inner side and outer side solutions, respectively. Firstly, 3D‐print a tube (inner diameter = 5.5 mm, outer diameter = 6 mm, length = 13 mm) in a circular container (inner diameter = 7.8 mm, depth = 15 mm) by 10% DBCO‐GelMA ensured that one end of the tube adhered tightly to the container. Next, the first ink was removed and a smaller tube (inner diameter = 5 mm, outer diameter = 5.5 mm, length = 13 mm) was 3D‐printed by the second ink (10% N3‐GelMA, 1% XUM‐MP‐1 or curcumin nanoparticles or nothing). Then, the inside and outside space of the double‐layer hydrogel had the same volume. 1 mL tween solution (1% wt%) was added in the inner side and outer side of the tube for drug release. The solution was collected and refreshed every day (from the first day to the seventh day). The collected incubation medium was stored in −20 °C container. This study was repeated three times, and the results were shown as mean value. The color shades of the solution showed the difference of curcumin concentrations. Concentrations of XMU‐MP‐1 were detected by ultraviolet spectrophotometry. The study of full wavelength scan displayed that the maximum absorption wavelength of XMU‐MP‐1 was near 303 nm. The absorption value of the collection solution at 303 nm was measured, and the absorption value XMP‐MP‐1 was obtained by subtracting the results of the experiment group from the blank group. The values of XMU‐MP‐1 content were calculated based on a standard curve.

##### Surgical of Nerves

Adult male SD rats (200–220 g) were used in this study. The experimental procedures were approved by the Institutional Animal Care and Use Committee of West China Hospital of Sichuan University and were performed in accordance with the guidelines and regulations of Sichuan University Committee on Animal Research and Ethics. The SD rats were divided into four groups: SADB group (*n* = 9), SABgroup (*n* = 9), ETE group (*n* = 9), and Sham group (*n* = 8). The animals were anesthetized with an intraperitoneal injection of chloral hydrate (0.3 mL/100 g). Then, the hair of the right leg was shaved after anesthetization. A skin incision of 30 mm was made and the sciatic nerve was exposed. For animals in SADB, SAB, and ETE groups, the right sciatic nerves were cut off. The end‐to‐end neurorrhaphy was performed with 8–0 absorbable vicryl sutures. The bandages with and without drug nanoparticles were attached, and the suture nerve was wrapped in SADB and SAB group, respectively. For all animals, the muscle and skin layers were closed with 2‐0 nylon sutures.

##### Statistical Analysis

All statistical analysis was conducted using Graphpad Prism 7 (Graphpad Software Inc.). The significance of drug release data was calculated using a Student's *t*‐test. The significance of other data was calculated using a one‐way ANOVA method. Results were displayed as means with standard deviation and *p* < 0.05 was considered as statistically significant.

## Conflict of Interest

The authors declare no conflict of interest.

## Supporting information

Supporting Information
