# Supplementary material for: A 3D‐Printed Self‐Adhesive Bandage with Drug Release for Peripheral Nerve Repair
Source: Adv Sci (Weinh). 2020 Oct 19;7(23):2002601. doi: 10.1002/advs.202002601 (PMC7709979; doi:10.1002/advs.202002601)
Supplement: Supplementary file 1 — Supporting Information [file ADVS-7-2002601-s001.pdf]

© 2020 Wiley-VCH GmbH

ADVANCED SCIENCE
Open Access

Supporting Information

for *Adv. Sci.,* DOI: 10.1002/advs.202002601

Energy Efficient Control of Ultrafast Spin Polarized Current to Induce Single Femtosecond Pulse Switching of a Ferromagnet

*Quentin Remy, Junta Igarashi, Satoshi Iihama, Grégory Malinowski, Michel Hehn, Jon Gorchon, Julius Hohlfeld, Shunsuke Fukami, Hideo Ohno, and Stéphane Mangin**

Supporting Information

A Self-adhesive Bandage Releasing Drug Directionally for Peripheral Nerve Repair

Jiumeng Zhang, Yuwen Chen, Yulan Huang, Wenbi Wu, Xianming Deng, Haofan Liu, Rong Li, Jie Tao, Xiang Li, Xuesong Liu, Maling Gou*

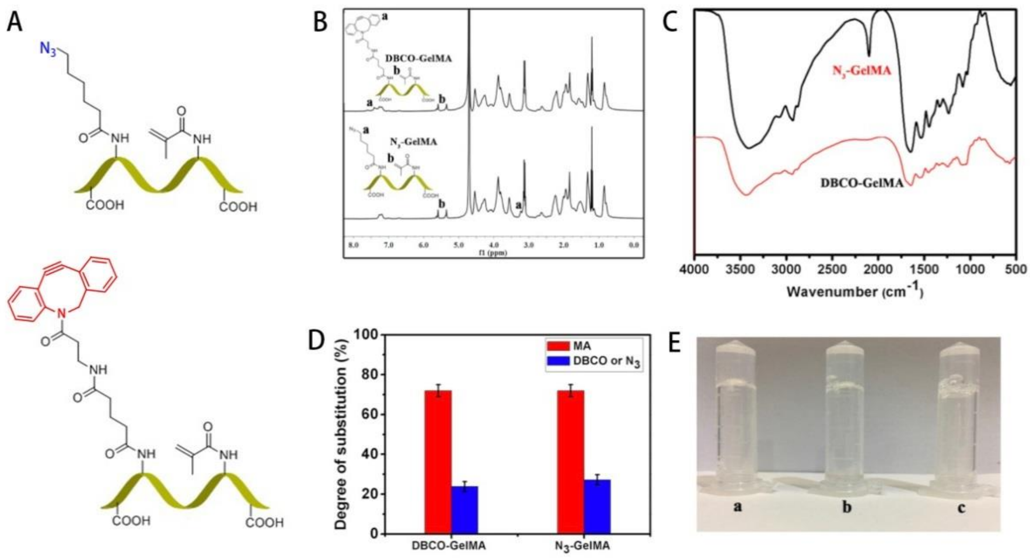

Figure S1. Synthesis and characterization of the set of photocrosslinkable clickable monomers. A) The chemical structures of the set of monomers. B), C) NMR and FTIR of the set of monomers. D) The substitution degree of MA and DBCO or N_3 to amino. These data are presented as mean ± standard deviation, n=3. E) a) and b) The photopolymerization N_3-GelMA and DBCO-GelMA respectively. c) The click crosslinking between N_3-GelMA and DBCO-GelMA.

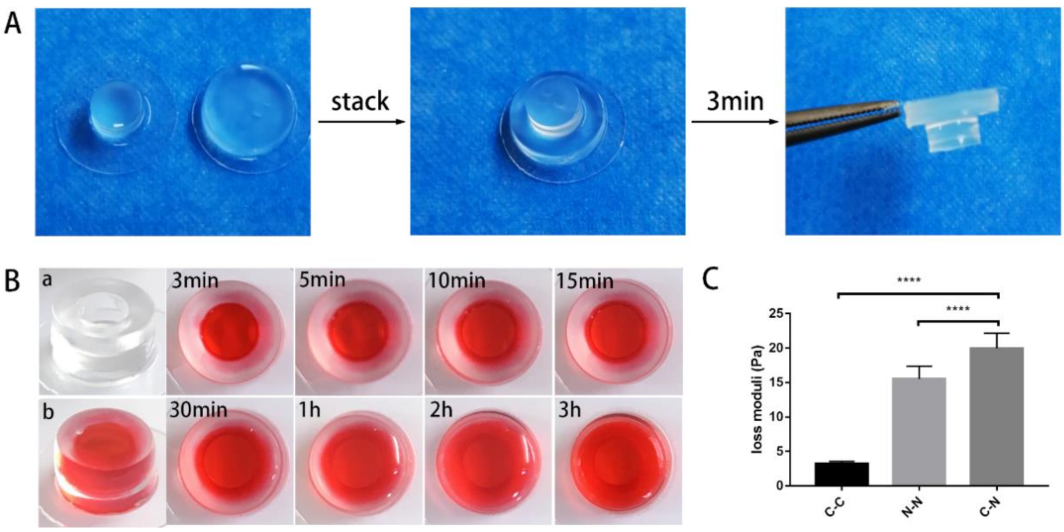

Figure S2. Self-adherive capacity between N_3-GelMA and DBCO-GelMA hydrogels. A) The adhering between N_3-GelMA and DBCO-GelMA hydrogel blocks. B) The leakproofness of a cup assembled by N_3-GelMA and DBCO-GelMA hydrogels. C) The loss modulus of samples assembled by N_3-GelMA and DBCO-GelMA hydrogels. These data are presented as mean ± standard deviation, n=3, one-way ANOVA, ****$p<0.0001$.

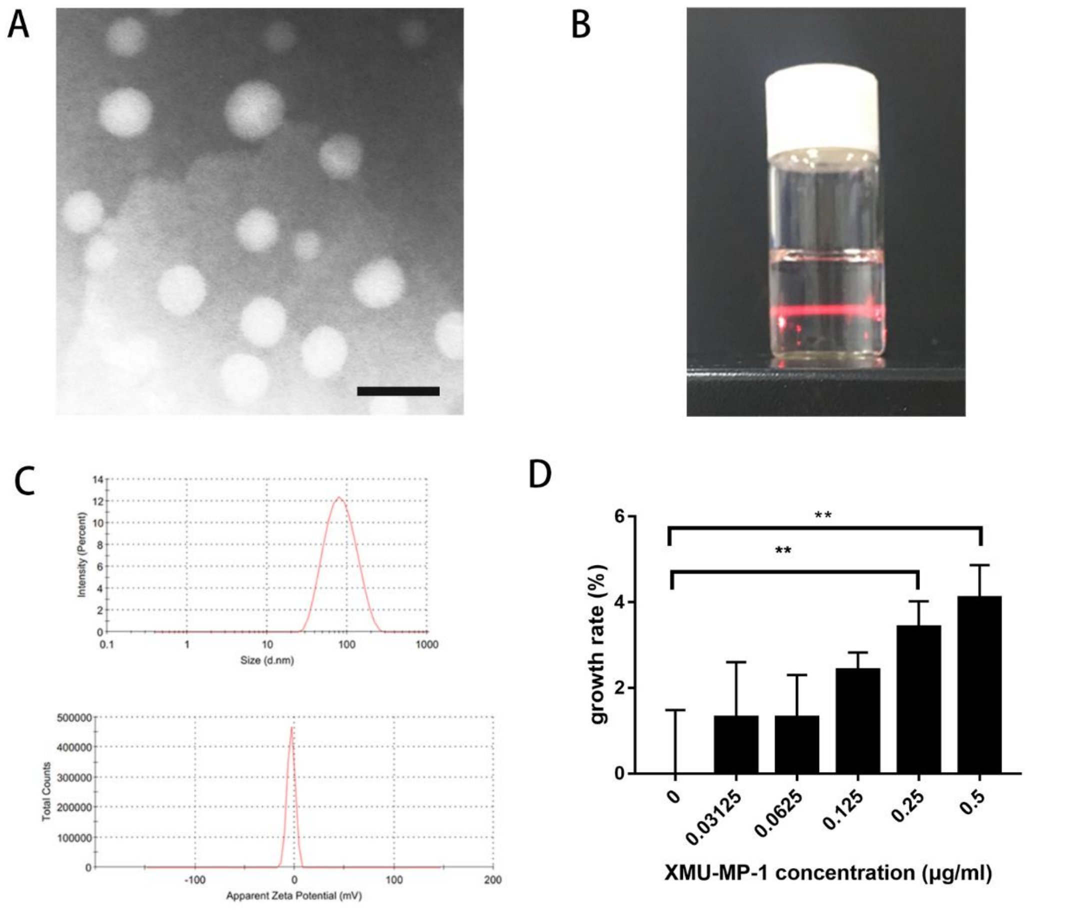

Figure S3. Preparation of XMU-MP-1 nanoparticles and curcumin release. A) TEM of the XMU-MP-1 nanoparticles (bar=100 μm). B) Tyndall Effect caused by the XMU-MP-1 nanoparticles. C) The mean zeta potential and size of the XMU-MP-1 nanoparticles. D) MTT results for cell proliferation of S16 at different XMU-MP-1 concentrations (0, 0.03125, 0.0625, 0.125, 0.25, 0.5 μg/ml). These data are presented as mean ± standard deviation, n=3, one-way ANOVA, **P<0.01.
